# Antimicrobial prescription patterns and ventilator associated pneumonia: findings from a 10-site prospective audit

**DOI:** 10.1186/s13104-018-3878-4

**Published:** 2018-10-29

**Authors:** Rosalind M. Elliott, Anthony R. Burrell, Peter W. Harrigan, Margherita Murgo, Kaye D. Rolls, David W. Sibbritt, Jonathan R. Iredell, Doug Elliott

**Affiliations:** 1 University of Technology Sydney, Broadway, NSW 2007 Australia; 2 Northern Sydney Local Health District, Reserve Road, St Leonards, NSW 2065 Australia; 3 John Hunter Hospital, Lookout Road, New Lambton, NSW 2305 Australia; 4 Australian Commission on Safety and Quality in Health Care, Sydney, 5/255 Elizabeth St, Sydney, NSW 2000 Australia; 5 The University of Sydney, Camperdown, NSW 2006 Australia; 6 Intensive Care NSW, Agency for Clinical Innovation, 67 Albert Avenue, Chatswood, NSW 2067 Australia; 7 Westmead Clinical School, The University of Sydney, Darcy Rd, Westmead, NSW 2145 Australia; 8 Infectious Diseases, Westmead Hospital and Western Sydney Local Health District, Darcy Rd, Westmead, NSW 2145 Australia; 9 Alliance for Vascular Access Teaching and Research, Menzies Health Institute, Griffith University, Meadowbrook, QLD 4222 Australia; 10 Faculty of Nursing and Midwifery, Sydney School of Nursing, The University of Sydney, Camperdown, NSW 2006 Australia

**Keywords:** Antibiotics, Antimicrobial stewardship, Incidence, Mechanical ventilation, Prescription, Prevalence, Surveillance, Ventilator-associated pneumonia

## Abstract

**Objective:**

To examine anti-microbial prescribing practices associated with ventilator-associated pneumonia from data gathered during an audit of practice and outcomes in intensive care units (ICUs) in a previously published study.

**Results:**

The patient sample of 169 was 65% male with an average age of 59.7 years, a mean APACHE II score of 20.6, and a median ICU stay of 11 days. While ventilator-associated pneumonia was identified using a specific 4-item checklist in 29 patients, agreement between the checklist and independent physician diagnosis was only 17%. Sputum microbe culture reporting was sparse. Approximately 75% of the sample was administered an antimicrobial (main indications: lung infection [54%] and prophylaxis [11%]). No clinical justification was documented for 20% of prescriptions. Piperacillin/tazobactam was most frequently prescribed (1/3rd of all antimicrobial prescriptions) with about half of those for prophylaxis. Variations in prescribing practices were identified, including apparent gaps in antimicrobial stewardship; particularly in relation to prescribing for prophylaxis and therapy de-escalation. Sputum microbe culture reports for VAP did not appear to contribute to prescribing decisions but physician suspicion of lung infection and empiric therapy rather than ventilator-associated pneumonia criteria and guideline concordance.

## Introduction

Reducing hospital-acquired infection is an important goal in improving quality of care and decreasing iatrogenic events for patients in hospital. Importantly, consistent and systematic information about ventilator-associated pneumonia-related (VAP) pathogens and associated antimicrobial prescribing practices in Australasian intensive care units (ICUs) is scarce. There are however some commonalities in pathogen types and prescribing patterns available from the international literature. Reports from Europe and North America suggest that microbes such as *Acinetobacter baumannii*, *Staphylococcus aureus* and members of the Enterobacteriaceae and *Pseudomonas* families are commonly associated with VAP [1]. Patterns of infection may vary over time and changes appear to be associated with antimicrobial use. For example, one European centre noted increased Enterobacteriaceae isolation rates (suggested by the authors to be related to antibiotic use), but unchanged *S. aureus* and *Pseudomonas aeruginosa* rates over a 5 years period [1].

Internationally, antimicrobial prescription rates are high in critical care, with prescriptions for VAP largely compliant with practice guidelines [2, 3]. Nevertheless some common areas have been identified for practice improvement in antimicrobial stewardship, including the use of culture-sensitive empiric therapy and appropriate de-escalation of therapy [4].

Given this context of an increasing incidence if antimicrobial resistance and recognition of the negative impact of hospital acquired infections, our aim was to develop a surveillance checklist for identification/screening of VAP and conduct an audit in a sample of ICUs in Australia and New Zealand to estimate the incidence of VAP. Audit data collected included antimicrobials prescribed and reports of microbial isolates in this cohort of mechanically ventilated patients.

The purpose of this brief research report is therefore to present previously unpublished data on antimicrobial prescription practices and offer our understanding of these practices in a sample of ICUs.

## Main text

### Methods

A prospective 30-day audit on clinical surveillance of VAP in 10 ICUs (9 in Australia, 1 in New Zealand) was conducted; 7 were tertiary referral units [5]. A more detailed report of the methods of the parent study was previously published [6]. Briefly, invitations to participate in the study were provided through mail distribution lists to Australian and New Zealand Intensive Care Society (ANZICS) members, with expressions of interest to participate received from medical directors of ICUs. Following institutional Review Board approval for each clinical site ICU-based research coordinators collected audit data for all patients: aged > 16 years; and mechanically ventilated (MV) for > 72 h.

Baseline data included age, gender, and diagnosis (at ICU admission). At or after 72 h of MV, data were collected daily using a case report form including a specific VAP checklist (decreasing gas exchange, sputum changes, chest X-ray infiltrates, inflammatory response; Table 1), reports of sputum collection for laboratory analysis (when ordered), microbes colonised (presence of microbes in the absence of disease)/grown (fungus, bacteria or virus) taken from microbiology laboratory reports, antimicrobial prescriptions for up to four medications each day, and independent ICU physician (intensivist) reports of VAP and infections, until ICU discharge. Day 30 survival outcome while in hospital was also recorded. A web-based database was used for data entry at each site. Descriptive data analysis is reported, using frequencies and proportions.

**Table 1:** Ventilator associated pneumonia (VAP) 4-item checklist

	Item	Definition
1	PaO_2_/FiO_2_ ratio^a^ ≤ 300 mmHg	Deterioration in gas exchange over last 24 h in the absence of cardiogenic pulmonary oedema or pulmonary disease
2	Sputum changes	A change in sputum characteristics, increased volume, or colour changes (yellow or green)
3	Chest X-ray infiltrates	New localised or diffuse infiltrates on a single Chest X-ray (not explained by cardiogenic pulmonary oedema or pulmonary disease)
4	Inflammatory response	≥ 1 of the following (in the absence of immunocompromise)
	a ↑ Temperature	New and persistent (last 24 h) elevated body temperature ≥ 38 °C (or > 37.5 °C if concurrent anti-pyretic medication administration)
	b WCC	White cell count ≤ 4 or ≥ 12 cells 10^9^/L for 2 days
	c ↑ Inflammation	Elevated serum inflammatory markers: C-reactive Protein (> 100 mg/L) or Procalcitonin (> 2.5 ng/L) for a single blood test

Three days after a patient is commenced on mechanical ventilation, are any of the following clinical items present?

*WCC* white cell count

^a^PaO_2_/FiO_2_ ratio: arterial oxygen tension divided by fraction of inspired oxygen

### Results

The demographic and clinical characteristics of the final sample of 169 patients are described in Table 2, along with a summary of VAP identification using the checklist and independent physician diagnosis, sputum findings and antimicrobial prescribing activities. There was a mean of eight data collection days per patient.

**Table 2:** Summary of audit findings

Characteristic		
Patient demographics (n = 169)		
Age (median—years)	59	
Sex—male (%)	65	
APACHE II (mean)	20.6	
Mechanical ventilation (median [IQR]—days)	7 [5–12]	
Length of stay (median—days)		
ICU	11	
Hospital	30	

	n/patient days	Antimicrobial prescribed^b^ n/patient days
VAP identified^a^		
Screening checklist	29/40	24 (83%)/34
Independent physician diagnosis	29/67	27 (93%)/60
Sputum findings		
Colonisation	31/41	36/41 (88%)
Infection^c^	51/94	64/94 (68%)
Screening checklist	13/13	
Independent physician diagnosis	9/10	

Antimicrobial prescriptions	%	
Antibiotic	90	
Antifungal	6	
Indication for prescription (%)	80	
Clinically-diagnosed lung infection	54	
Prophylaxis	11	
Bloodstream infection	9	

^a^Predominantly different sets of patients; five patients were classified with VAP using both methods

^b^All patients classified with VAP using both methods received antimicrobials

^c^Most frequently identified microbes: ‘Other Gram negatives’ and candida; ‘*Pseudomonas* sp.’; ‘*Coagulase*-*negative staphylococcus*’

Of note, antimicrobial agents were prescribed in the absence of abnormal sputum findings for 73% of the data collection days. For VAP cases identified using the screening checklist, antimicrobials were prescribed for 83% of patients, despite limited reporting of colonised or infected sputum (from microbiology reports). Piperacillin/tazobactam comprised 32% of antimicrobial prescriptions. The main prophylaxis antimicrobials were cefazolin (12 patients, mean 3 days), and piperacillin/tazobactam (10 patients, mean 3 days); acyclovir was also prescribed (6 patients, mean 4 days). No agents were prescribed simultaneously.

### Discussion

Three key findings are noted from this microbial-focused report of the audit: (1) sputum collection for microbiological culture and sensitivity testing were rarely requested, and appeared irrelevant for prescribing practices in this sample; (2) antibiotics appeared to be commonly prescribed for prophylaxis; and (3) treating physicians appeared to diagnose VAP and prescribe antimicrobials based on clinical assessment, both independent of the clinical signs reflected in the VAP checklist and any available microbial reports.

While reports of sputum microbe isolates in patients classified with VAP in this cohort were sparse, a number of microbes associated with VAP were identified. *Pseudomonas*, *Haemophilus*, Methicillin sensitive *Staphylococcus aureus* (MSSA), Methicillin resistant *Staphylococcus aureus* (MRSA) and *Escherichia coli* are commonly reported in the literature [7]. Antimicrobial prescriptions were appropriate when sputum isolates were identified.

Prescribing practices may be influenced by different reporting practices and language in microbiological reporting. For example, laboratory reporting species-level identification and/or antibiotic sensitivities may lead to increased antibiotic prescriptions. Also of note, antimicrobial prescription rates were 20% higher for patients with colonised sputum compared to infected sputum. It is known that many clinicians consider ‘colonisation’ as the beginning of microbial infection; only a few consider colonisation and infection as different processes. It appears that physicians relied on their clinical judgement when prescribing antimicrobials.

Pulmonary infection accounted for just over half of all antimicrobial prescriptions in this sample; 10% lower than international estimates of the prevalence of infection types in ICU (64%) [8]. Given the study design, we were unable to examine physician considerations of factors known to affect treatment effectiveness (e.g. previous antimicrobial exposure; antibiogram for each setting). Considering local antibiograms is now highly recommended when prescribing antimicrobials for nosocomial pulmonary infections to reduce the incidence of resistant organisms [9].

The rate of antimicrobials prescribed as an apparent prophylaxis was high (11%), given that our inclusion criteria likely excluded the majority of patients treated in ICU for postoperative care. The duration of treatment and types of antimicrobials (i.e. cefazolin and piperacillin/tazobactam) prescribed for prophylaxis was of concern, given that a single only antimicrobial dose is recommended for the majority of surgeries. One possible explanation for this was the use of empiric therapy; physicians suspected, but could not confirm, a respiratory infection; or were attempting to prevent pneumonia within the setting of immunosuppression (we did not collect data about immune status).

Importantly, while one in 10 prescriptions for prophylaxis is reflective of prescribing practices in Australian hospitals, this is double the target of 5% set by the national peak body [10]. This rate may however be reflective of prescribing practices in ICU, where rates are approximately twice those found in other hospital settings [10]. It would also appear that de-escalation of antimicrobial therapy was not extensively practiced, given the duration of broad spectrum antibiotic therapy. Prescription rates in Australia are among the highest in the developed World, so practices identified here may be reflective of overall health care practice in the country [10].

Antimicrobials were prescribed for more days for patients with a physician-diagnosis of VAP (compared to the VAP checklist). This is a logical finding, given that once a physician diagnosed (and documented) the presence of pneumonia, specific treatment would follow. Interestingly, the type of antimicrobial prescribed for patients with possible VAP using the screening checklist (e.g. Gram-negative antibiotics with anti-pseudomonal activity) suggested that treatment was focused on a pulmonary infection.

Isolated sputum microbes were different for the two methods of VAP ‘diagnosis’ or ‘identification’ (noting that only five patients [17%] were classified using both methods). Given the small number of patients classified as having VAP, potential reasons for this variation cannot be elucidated; it is however unlikely that clinical differences such as patient ICU admission diagnosis and severity of illness were influencing factors [6].

This audit of 10 ICUs in Australia and New Zealand identified variations in antimicrobial prescribing practice in the context of VAP. Ordering of sputum microbial isolates was rare, and therefore the contribution of these reports to prescribing decisions was not evident, given the frequency of antimicrobial prescriptions for lung infection. It therefore appears that prescribing decisions were based on clinician suspicion of an infective lung process, and empiric therapy rather than the use of identified VAP criteria and guideline concordance. From an antimicrobial stewardship perspective, opportunities for reflections on and improvements in practice are evident, including reducing the prevalence of prescribing for prophylaxis, and de-escalation of antimicrobial treatment according to accepted practice guidelines and recent expert recommendations [3].

## Limitations

From a methodological perspective, the audit design enabled sampling from multiple sites using a consistent, standardised data collection approach. Most study ICUs were however large tertiary-referral units, potentially limiting the representativeness of this sample to the broader ICU population, particularly for different countries and health systems. A limitation of using independent assessors in data collection was that the real-time, decision-making processes of physicians during their independent diagnosis of VAP and/or their anti-microbial prescribing practices remains unknown. Our interpretations are therefore based on objective clinical diagnostic and microbiological data collected during the audit. We did not also collect data about the individual ICU contexts, specifically their antimicrobial policies (e.g. stewardship) and local antibiograms at the time data were collected.

## Abbreviations

APACHE II: acute physiology and chronic health evaluation II
FiO_2_: fraction of inspired oxygen
ICU: intensive care unit
MRO: multiresistant organism
MRSA: methicillin resistant *Staphylococcus aureus*
MSSA: methicillin sensitive *Staphylococcus aureus*
MV: mechanical ventilation
PaO_2_: partial pressure of oxygen
VAP: ventilator associated pneumonia
WCC: white cell count
